# Cost-Sharing Rates Increase During Deep Recession: Preliminary Data From Greece

**DOI:** 10.15171/ijhpm.2016.62

**Published:** 2016-05-28

**Authors:** Athanasios Gouvalas, Michael Igoumenidis, Mamas Theodorou, Kostas Athanasakis

**Affiliations:** ^1^Pharmaceutical Association of Fthiotida Prefecture, Lamia, Greece.; ^2^Technological Educational Institute of Western Greece, Faculty of Nursing, Patra, Greece.; ^3^Open University of Cyprus, Latsia, Cyprus.; ^4^National School of Public Health, Open University of Cyprus, Latsia, Cyprus.

**Keywords:** Cost-Sharing, Patient Participation, Prescription Drugs, Out-of-Pocket (OOP) Expenses

## Abstract

**Background:** Measures taken over the past four years in Greece to reduce pharmaceutical expenditure have led to significant price reductions for medicines, but have also changed patient cost-sharing rates for prescription drugs. This study attempts to capture the resulting increase in patients’ out-of-pocket (OOP) expenses for prescription drugs during the 2011-2014 period.

**Methods:** The authors conducted a retrospective review of financial data derived from 39 883 prescriptions, dispensed at three randomly chosen pharmacies located in Lamia, central Greece.

**Results:** The study recorded an average contribution rate per prescription as follows: 11.28% for 2011 (95% CI: 10.76-11.80), 14.10% for 2012, 19.97% for 2013, and 29.08% for 2014. Correspondingly, the mean patient charge per prescription for 2011 was €6.58 (95% CI: 6.22-6.94), €8.28 for 2012, €8.35 for 2013, and €10.87 for 2014. During the 2011-2014 period, mean percentage rate of patient contribution increased by 157.75%, while average patient charge per prescription in current prices increased by 65.22%. The use of a newly introduced internal reference price (IRP) system increased the level of prescription charge at a rate of 2.41% for 2012 (100% surcharge on patients), 26.24% for 2013 (49.95% on patients and 50.04% on the appropriate health insurance funds), and 47.72% for 2014 (85.06% on patients and 14.94% on funds).

**Conclusion:** Increased cost-sharing rates for prescription drugs can reduce public pharmaceutical expenditure, but international experience shows that rising OOP expenses can compromise patients’ ability to pay, particularly when it comes to chronic diseases and vulnerable populations. Various suggestions could be effective in refining the cost-sharing approach by giving greater consideration to chronic patients, and to the poor and elderly.

## Background

The ongoing financial crisis in Greece has already led to drastic changes in the country’s health sector. Total health expenditure fell from 10.1% of the gross domestic product (GDP) in 2009^1^ to 6% in 2014.^2^ Apart from other health reforms, pharmaceutical expenses have been downsized from 2.3% of the GDP in 2012 to less than 1% in 2014, below the European Union (EU) average of 1.5%.^3^ This reduction was a priority for the government, and it was mandated by specific macroeconomic indicators; Greece had one of the highest rates of pharmaceutical expenditure among The Organization for Economic Co-operation and Development (OECD) countries in 2012 (25.2% as a percentage of health expenditure compared to the OECD average of 15.9%),^3^ whereas public funding for pharmaceuticals was set at 1.5% of the GDP in Greece for 2012, which amounted to the highest percentage among EU countries.^4^ Among others, the government’s interventions included drug re-pricing, policies to promote the use of generics, and changes in the country’s prescription drug cost-sharing policy.^5^

With respect to cost-sharing, the Greek system functions based on three percentage classes, namely 25%, 10%, and 0%. This means that, depending on the percentage class where each disease is classified, the patients face out-of-pocket (OOP) expenses corresponding to 25%, 10%, or 0% of the price of the drugs which the physicians prescribe for the diseases. Prescribing physicians must be affiliated with the National Organization for Healthcare Provision (EOPYY) – the country’s major health insurance fund – which covers the rest of the amount (75%, 90%, or 100%), and the patients must be registered with EOPYY, either as pensioners or as insured active workers – the latter pay monthly premiums that are normally proportional to their income. Uninsured patients have to pay the whole price of their drugs themselves, unless they are poor (annual income below €5000) and unemployed; in that case, they are entitled to free drugs for specific conditions (various restrictions apply) until they are employed and, thus, become insured. Once they have their physician’s prescription, patients are free to choose any community dispensing pharmacy to dispense it by paying accordingly 25%, 10%, or 0% of the drug’s price to the pharmacist. The recent government reforms re-classified diseases and their corresponding drugs, by shifting more diseases to the highest cost-sharing class of 25%,^6,7^ and by leaving some chronic conditions to the class of 10% and some life-threatening conditions to the class of 0%.^8^ In essence, this means that very few patients are left with free prescription drugs. End-stage chronic renal failure patients and paraplegics are amongst those who still get their drugs for free, whereas particular exceptions apply to very limited populations, such as beneficiaries of state relief funds – that is, elderly pensioners below poverty level.

In addition, the government introduced a new internal reference price (IRP) calculation system in an effort to further contain the cost of pharmaceutical care.^9,10^ The IRP is set by Social Security according to therapeutic categories, and, as of 2014 all chemicals and preparations are subject to the IRP system. This leads to more OOP expenses for the patient; apart from paying 10% or 25% of the drug IRP, the patient also has to pay the price difference in case that the drug retail price is higher than the IRP.

Considering the above, the challenge for policy-makers is to determine whether these measures are effective, by achieving a fair allocation of the burden of pharmaceutical expenditure at the same time. From 2009 and onwards, public pharmaceutical expenditure followed a downward trend, from approximately €5.3 billion in 2009 to approximately €2 billion in 2014^11^; however, a substantial part of this important fiscal adjustment is attributed to the patients’ increased OOP expenses under the current cost-sharing scheme. The aim of this paper is to present the evolution of these expenses between 2011 and 2014, that is, the main period in which major interventions in the pharmaceutical sector took place.

## Methods

### Design and Sampling

We performed a retrospective analysis of financial data related to medical prescriptions dispensed at three randomly chosen community pharmacies. These pharmacies were located in the municipality of Lamia, capital city of the prefecture of Fthiotida in Central Greece. For the purpose of the study, dispensing pharmacists gave us permission to access their electronic records by using their professional software. In order to take into account the seasonal nature of drug prescribing, we chose to examine the following time intervals: 1/5/2011-31/8/2011, 1/5/2012-31/8/2012, 1/5/2013-31/8/2013, and 1/5/2014-31/8/2014. Thus, we were able to compare similar periods of different years. During the sample collection process we used only financial data deemed necessary for this study, and in accordance with Law 2472/97 on the Protection of Individuals regarding the Processing of Personal Data. Microsoft Excel 2007 software was used to consolidate all data collected, and SPSS 20.0 was used for the statistical analysis.

With regard to the year 2011, we used data consolidated from various health and social security funds. This was mandated by the high level of fragmentation that existed within health insurance before the 2012 reform, as there used to be 108 different health and social security funds in the public sector. Since 2012, a main fund (EOPYY) essentially integrates all the other funds and handles all medical prescriptions, thus, rendering data processing much easier.

We excluded from the sample prescriptions related to high cost medicinal products for the treatment of rare diseases, due to changes in the distribution channels of these products that coincided with the period of research. Overall, 10 421 prescriptions were collected for 2011, 8964 prescriptions for 2012, 9391 prescriptions for 2013, and 11 107 prescriptions for 2014, reaching a total sample size of 39 883 prescriptions.

### Calculations

We calculated mean prescription cost-sharing values per year in a percentage form and at current € prices. Subsequently, we calculated percentage changes and price changes between years 2012 and 2011, 2013 and 2012, 2014 and 2013, as well as 2014 and 2011. In addition, we calculated the average price per prescription per year, set at €6.58 for 2011 (95% CI: 6.17-6.99), €8.28 (95% CI: 7.59 to 8.97) for 2012, €8.35 (95% CI: 7.59-9.11) for 2013, and €10.87 (95% CI: 9.71-12.03) for 2014.

Concerning the aforementioned IRP system, we measured its percentage contribution to the shaping of the patients’ OOP expenses separately for each year. Then we estimated the distribution of this percentage between EOPYY and patients for the corresponding time intervals, based on the differing cost-sharing rates for each drug category and each year. For the year 2011, there was no difference between the drug retail price and the IRP (the new IRP system had not been introduced yet). For the year 2012, the price difference concerned only 10 active substances (it was the new system’s trial period), and the cost burden fell entirely upon the patients. For the year 2013, the price difference concerned all branded drugs with generic counterparts in the market, and it was shared equally between patients and EOPYY. For the year 2014, the price difference concerned all substances under the following provisions: for drugs with a higher retail price than the IRP, the difference is covered entirely by the patient; if these drugs have no generic counterpart, the difference is equally shared between patients and EOPYY; and finally, for drugs with no generic counterpart and with a lower retail price than the IRP, the difference is deducted from the anticipated institutionalized patient contribution at half-maximum.^12,13^ We also added a fixed fee of €1 per prescription (to be paid by the patient), which was introduced that year as a measure to control demand.

## Results

Table 1 summarizes the results of the data analysis in a cumulative format (95% CI), regarding the evolution of patients’ OOP expenses over time. Considering the 2011-2014 period as a whole, there was a cumulative rise of patient participation rate by 157.75%.

**Table 1:** OOP Expenses and Related Changes, 2011-2014

**Year**	**No. of Prescriptions**	**OOP Expenses (%)**	**OOP Expense/Prescription (€)**	**OOP Expenses Change (%)**	**Price Difference (%)**	**Average Price per Prescription (€)**
2011	10 421	11.28	6.58	‏-	‏-	58.29
2012	8964	14.10	8.28	24.94	25.90	58.73
2013	9391	19.97	8.35	41.63	0.90	41.84
2014	11 107	29.08	10.87	45.66	30.05	37.36
Total	39 883	‏-	‏-	157.75	65.22	‏-

Abbreviation: OOP, out-of-pocket.

Table 2 presents the effect of the IRP system on OOP expenses, which was pilot tested in 2012 (with patients bearing the full cost for 10 selected active substances) and gradually expanded as explained above. In 2014, the IRP system contributed to patients’ OOP expenses by 47.72%.

**Table 2:** Effect of the IRP System on Total Patient Contribution, 2011-2014

**Year**	**No. of Prescriptions**	**Impact of IRP System in OOP Expenses Formulation (%)**	**EOPPY Allocation (%)**	**Patient Allocation (%)**
2011	10 421	-	-	-
2012	8964	2.41	0.00	100.00
2013	9391	26.24	50.04	49.95
2014	11 107	47.72	14.96	85.06

Abbreviations: OOP, out-of-pocket; IRP, internal reference price; EOPYY, National Organization for Healthcare Provision.

## Discussion

The results presented above indicate a rising OOP burden for pharmaceutical care in Greece, which can be directly attributed to increased cost-sharing rates and the new IRP system. Prescription flow does not show any significant or consistent changes between different years, as the number of prescriptions is reduced for the year 2012 and then it rises again, exceeding in 2014 the numbers of 2011. The results show that the government has succeeded in reducing public pharmaceutical expenditure mainly by shifting more of the cost to care recipients. Achieving fiscal targets is crucial to the country’s effort to overcome recession, but caution is warranted to avoid negative consequences that enhance the public’s perception that policy reforms are unfair. Change in cost-sharing rates and reference pricing constitute widely implemented policies when trying to reduce pharmaceutical expenditure, and they have been part of many recent health policy reforms internationally.^14^ Clinical and economic outcomes of such policies are difficult to assess; however, relevant bibliography suggests that significant implications may occur. The main challenge is to rationalize the use of drugs in a cost-effective manner, without compromising the public’s health.

In general, medication adherence tends to be compromised when cost-sharing schemes result to increases in OOP expenses.^15^ Recent systematic reviews show that increased cost-sharing policies entail the possibility of reductions in the use of life-sustaining drugs, as well as drugs that are important in treating chronic conditions.^16-18^ Reduced medication adherence (taking fewer doses, postponing taking a medication, failing to fill a prescription at all, and taking medication less frequently than prescribed) causes serious adverse events, especially for vulnerable populations such as the poor and the elderly.^19,20^ In Italy, a country also hit by economic recession, a recent analysis suggests that changes in cost-sharing policies were partly successful, in terms of greater revenue to the health system, but in the last few years, cost-sharing increases would seem to have rebounded negatively on more vulnerable families, due to the economic crisis.^21^ In Spain, where a set of cost-sharing reforms on pharmaceutical prescriptions with regional variants have been established since July 2012, in the context of heavy austerity reforms of public financing, a dramatic reduction in the use of drugs has already been noted.^22^ Even in countries without serious financial problems, such as Sweden or Australia, large increases in cost-sharing impact on patients’ ability to afford essential drugs.^23,24^

There is not yet a large study in Greece from which to deduct similar conclusions, but a few studies confirm that patients’ ability to pay has been compromised, with potentially serious adverse events. For instance, Tsiligianni et al report that many chronic patients have begun to reduce or skip doses of their prescription medicines,^25^ whereas Skroumpelos et al estimate that increases greater than 50% in chronic patients’ participation to medical cost leads to adverse health outcomes for more than one-third of these patients.^26^ More studies are needed in order to properly assess the overall impact of these policy changes, and to determine whether positive consequences outweigh negative ones. For instance, excessive drug use is a well-documented problem in Greece,^27^ which could be alleviated under the current cost-sharing scheme. However, policy reforms should keep balance between cutting unnecessary expenses and protecting vulnerable populations.

### Proposals

Literature abounds with suggestions on how to refine cost-sharing policies in order to achieve better results. An analysis of the impact of cost-sharing changes on the demand for 8 classes of prescription drugs shows that the influence of cost-sharing on drug use may be related to characteristics inherent to each drug class or underlying condition.^28^ This means that the Greek approach, based solely on three cost-sharing categories of 25%, 10%, and 0%, is oversimplified. More categories are needed, depending on the underlying diseases. Hossein and Gerrard analyzed trends in the United Kingdom, Germany, Japan, France, and the United States, and found that, in spite of higher levels of cost-sharing, OOP spending as a percentage of total spending remained unchanged in most of these countries because they instituted programs to protect certain categories of individuals by creating OOP limits, exempting people with certain chronic diseases, or eliminating cost-sharing for certain demographic groups and low-income people.^29^ These refinements are very limited in Greece, and they need to be carefully examined. Cohn suggests a blended approach, citing the French healthcare system, in which OOP spending is reduced for the sick and the poor, by waiving cost-sharing for chronic conditions.^30^ Riggs and Ubel emphasize on the role of prescribing physicians, who can help to ensure that their patients achieve the best financial and medical outcome, by helping them navigate trade-offs related to their OOP costs.^31^

In Cyprus, a country with close geographical and cultural proximity to Greece, major healthcare reforms are currently taking place as a response to financial crisis, and experts design pharmaceutical policies which should not compromise access to or quality of treatment.^32^ Petrou warns that the government must resist the temptation to further increase co-payment, as it disproportionately burdens people with lower income or more healthcare needs, and he proposes safeguarding of access for vulnerable groups through exceptions, setting of a maximum ceiling for co-payment, and the introduction of a variable co-payment, contingent to patient’s income.^33^ In a different direction, Petrou and Vandoros propose tendering for pharmaceuticals as a sustainable and context sensitive cost containment approach,^34^ which could also be an option for the Greek government. Also, a recent Cypriot survey with semi-structured interviews concludes that the country will need to increase the market share of generic medicines to contain drug spending.^35^ This is also an area of reform lagging behind in Greece. Recent studies suggest that Greek patients are sceptical with the use of generic drugs, questioning their safety and effectiveness.^36^ Therefore, the government needs to promote the use of generic medicines by proper education and access to reliable information, and by further discounts or even zero-cost patient contribution when a generic drug is chosen.

Another country whose experience could be valuable for Greece is Ireland. Ireland and Greece went through very similar economic trials, both receiving bailouts and both having to change public spending due to their creditors’ demands. In addition, Ireland is similar to Greece in terms of pharmaceutical expenditure: in 2009, the level of public spending for pharmaceuticals in Ireland was exceeded only by Greece, Canada, and the United States, among OECD countries.^37^ When Ireland started implementing cost containment strategies, a €0.50 copayment per prescription item (capped at €10 per household per month) was introduced on the national tax-funded health insurance program for low-income individuals and older people, which ended free access to prescription medicines.^38^ Sinnott et al remark that this small co-payment had little impact on adherence for essential medicines, and a potentially significant reduction in use of less-essential medicines that were overprescribed before the co-payment – they do note. However, there are exceptions and we need a better understanding of the clinical consequences of reductions in use of essential medicines, even if these reductions are small.^38^ Ireland has also achieved high compliance with policies related to generic drug use, with researchers mentioning enactment of supportive legislation, acceptability of active-substance based generic substitution and the phased nature of the policy introduction as success factors.^39^ Greek policy-makers should explore in detail the measures introduced in Ireland, gain insight, and possibly co-operate with their Irish counterparts in order to contain pharmaceutical costs.

All the above mentioned proposals could be useful in the direction of achieving fiscal targets without burdening vulnerable populations. However, before all else, an important structural problem has to be overcome. Before entering into deep recession, Greece had been slow and inefficient in making necessary reforms to contain public expenditures, and pharmaceutical expenditure amongst them.^40^ For instance, both a 2008 presidential decree^41^ and a 2010 state law^42^ with regards to prescribing physicians’ and dispensing pharmacists’ responsibilities seem to forbid generic substitution. Since 2010, when the country went essentially bankrupt, successive governments have been trying to reform Greece’s economy by implementing a variety of measures. These measures have not always been properly discussed, as the country’s creditors set strict deadlines for their implementation. The new cost-sharing scheme and the new IRP system are no exceptions. They have to be further examined, and policy-makers need to consider expert opinions, economic analyses, international experience, and patients’ needs, especially for vulnerable populations, before making all necessary amendments that the creditors should allow. As the Council of Europe suggests, new policies related to pharmaceutical expenses need to be flexible and constantly subject to revision; that is, they have to be able to change drastically if it can be shown that their adverse effects in health outweigh financial benefits.^43^ This can be achieved with investment in research to identify which policies can cut expenses without unintended consequences,^44^ and with the exploration of as many alternative options as possible instead of hasty changes in cost-sharing procedures.^45^ Cost-sharing can work wonders, but only in the right dosage and as part of a broader treatment plan.^30^

## Limitations

This study is subject to certain limitations. First, it was conducted in a single region of Greece and included prescription samples from only three pharmacies. Although there is no reason to expect that prescription flow would show major differences in other geographical areas, the relatively small sample remains a disadvantage. In addition, we systematically excluded high-cost medicinal products for the treatment of rare diseases from our study, as they might have biased the findings, due to changes in the distribution channels of these products that coincided with the period of research. Finally, another limitation is that we were not able to link increases in cost-sharing with reductions in drug utilization, as the number of prescriptions is not greatly affected overall. However, this is something that cannot be excluded in the long run, or with a bigger sample. It is expected that this study shall be used as a basis for more large-scale studies in the future, when positive and negative aspects of recent reforms shall become more apparent.

## Conclusion

Cost-sharing reforms that were recently implemented in Greece resulted in significant increases of OOP payments. We suggest that, in order to maintain criteria of equitable burden sharing and efficient use of resources, patients’ contributions must be proportional to income – the poor and the elderly cost-sharing ratio should be lower – and inversely proportional to health needs – chronic patients should pay less depending on the severity of their situation. Greek policy-makers should be given the opportunity to carefully assess all the other viable options regarding pharmaceutical cost containment, and adjust cost-sharing schemes to meet the needs of vulnerable populations.

## Competing interests

Authors declare that they have no competing interests.

## Ethical issues

This study was approved by the Ethics Committee of Pharmaceutical Association of Fthiotida, Lamia, Greece. During the prescription data processing, there was no access to names or other characteristics of the corresponding patients.

## Authors’ contributions

AG: Contribution to the conception and design of the work, contribution to study implementation and data gathering, data analysis and providing the manuscript. MI: Contribution in the analysis, and interpretation of data, and the critical revision. MT: Contribution to data analysis and providing the manuscript. KA: Contribution to data analysis and providing the manuscript.

## Authors’ affiliations

^1^Pharmaceutical Association of Fthiotida Prefecture, Lamia, Greece. ^2^Technological Educational Institute of Western Greece, Faculty of Nursing, Patra, Greece. ^3^Open University of Cyprus, Latsia, Cyprus. ^4^National School of Public Health, Open University of Cyprus, Latsia, Cyprus.

## Key messages

**Implications for policy makers**

- Policy-makers should take into account proportionality of income along with patient needs – the poor and the elderly need to be protected.
- Policy-makers should promote the use of generic drugs, by examining failed attempts of the past and by implementing policies proven to be effective in countries with similar characteristics to Greece.
- The cost-sharing mechanism needs to be flexible and subject to changes depending on its positive and negative results over time.

**Implications for public**

Structures and mechanisms need to be put in place to enable awareness of community about cost-sharing through a system of integrity and transparency. Adherence to pharmacological treatment has to be monitored in a consistent way in order to assess long-term effects of policy changes to the public’s health.
